# Identification of fibroblast-related genes based on single-cell and machine learning to predict the prognosis and endocrine metabolism of pancreatic cancer

**DOI:** 10.3389/fendo.2023.1201755

**Published:** 2023-07-31

**Authors:** Yinghua Xu, Xionghuan Chen, Nan Liu, Zhong Chu, Qiang Wang

**Affiliations:** ^1^Department of Translational Medicine and Clinical Research, Sir Run Run Shaw Hospital, Zhejiang University School of Medicine, Hangzhou, China; ^2^Department of General Surgery, Sir Run Run Shaw Hospital, Zhejiang University School of Medicine, Hangzhou, China; ^3^Department of Trauma Surgery, Tiantai People’s Hospital of Zhejiang Province, Taizhou, China

**Keywords:** single-cell sequencing, pancreatic adenocarcinoma, tumorigenesis, RiskScore, prognosis, endocrine metabolism

## Abstract

**Background:**

Single-cell sequencing technology has become an indispensable tool in tumor mechanism and heterogeneity studies. Pancreatic adenocarcinoma (PAAD) lacks early specific symptoms, and comprehensive bioinformatics analysis for PAAD contributes to the developmental mechanisms.

**Methods:**

We performed dimensionality reduction analysis on the single-cell sequencing data GSE165399 of PAAD to obtain the specific cell clusters. We then obtained cell cluster-associated gene modules by weighted co-expression network analysis and identified tumorigenesis-associated cell clusters and gene modules in PAAD by trajectory analysis. Tumor-associated genes of PAAD were intersected with cell cluster marker genes and within the signature module to obtain genes associated with PAAD occurrence to construct a prognostic risk assessment tool by the COX model. The performance of the model was assessed by the Kaplan–Meier (K-M) curve and the receiver operating characteristic (ROC) curve. The score of endocrine pathways was assessed by ssGSEA analysis.

**Results:**

The PAAD single-cell dataset GSE165399 was filtered and downscaled, and finally, 17 cell subgroups were filtered and 17 cell clusters were labeled. WGCNA analysis revealed that the brown module was most associated with tumorigenesis. Among them, the brown module was significantly associated with C11 and C14 cell clusters. C11 and C14 cell clusters belonged to fibroblast and circulating fetal cells, respectively, and trajectory analysis showed low heterogeneity for fibroblast and extremely high heterogeneity for circulating fetal cells. Next, through differential analysis, we found that genes within the C11 cluster were highly associated with tumorigenesis. Finally, we constructed the RiskScore system, and K-M curves and ROC curves revealed that RiskScore possessed objective clinical prognostic potential and demonstrated consistent robustness in multiple datasets. The low-risk group presented a higher endocrine metabolism and lower immune infiltrate state.

**Conclusion:**

We identified prognostic models consisting of APOL1, BHLHE40, CLMP, GNG12, LOX, LY6E, MYL12B, RND3, SOX4, and RiskScore showed promising clinical value. RiskScore possibly carries a credible clinical prognostic potential for PAAD.

## Introduction

Although pancreatic adenocarcinoma (PAAD) is a relatively low-incidence cancer, it is a highly lethal tumor (1). The deficiency of specific early symptoms of PAAD and the fact that the majority of patients are experiencing advanced progression or organ metastases contribute to PAAD being a high-mortality cancer (2). Frustratingly, radiotherapy, as well as chemotherapy, were not effective options in the treatment of PAAD, and surgical resection was currently the best option for most patients, but the prognosis was graded poorly, with an overall 5-year survival (OS) rate of less than 10% (3–5). Several studies have shown that prognosis in a variety of cancers, including PAAD, can be predicted using carbohydrate antigen 19-9 (CA 19-9) and carcinoembryonic antigen (CEA) (6, 7). However, they lack specificity and sensitivity for PAAD (8). To address the clinical pain point that PAAD prognosis was difficult to assess, it was imperative to develop effective prognostic tools to achieve patient prognostic risk assessment as well as personalized and precise treatment.

The pancreas has two functions: endocrine and exocrine. The exocrine glands of the pancreas are composed of acinar cells and duct cells. Previous studies have believed that pancreatic ductal adenocarcinoma (PDAC) originates from ductal cells because of tumor histological similarity to ductal morphology (9). On the other hand, pancreatic endocrine tumors are caused by endocrine cells (10). With the emergence of single-cell RNA sequencing (scRNA-Seq) technology, exploring deeper molecular mechanisms of life from cellular genetic material, functional heterogeneity, and the identification of specific cell subtypes emerged as mainstream research directions (11, 12). scRNA-seq maps the gene expression patterns of each cell and decodes its intercellular signaling network. This unbiased characterization provides clear insights into the entire tumor ecosystem, such as the mechanisms of intra- and intertumor heterogeneity and the tumor microenvironment (13). Tumors lead to an individualized prognosis and variable therapeutic responses due to their heterogeneity, and single-cell technologies showed powerful functions in revealing the molecular mechanisms of cancer through the precise analysis of specific cells or cell clusters (14–16). For example, Wang et al. developed a lung cancer artificial intelligence detector using scRNA-Seq data from early lung cancer, which showed great specificity in the early detection of lung cancer and large-scale early screening of high-risk populations (17). Li et al. identified proinvasive cancer-associated fibroblast subtypes in patients with poor prognosis for gastric cancer, and inhibition of these cell subsets contributed to creating an activated immune tumor microenvironment (TME) (18). These studies demonstrated the ability to integrate scRNA-Seq data to deepen insights into cancer.

Fibroblast growth in pancreatic cancer (PDAC) tumors is known as a tumor suppressor (19, 20). Cancer-associated fibroblasts (CAFs) are a collective term for these cells. CAFs may play a role in the development and progression of PDAC and the response to treatment (21, 22). CAFs are an important stromal component, secreting growth factors, inflammation mediators, and extracellular matrix (ECM) proteins that facilitate tumor growth, resistance to therapy, and immune rejection (23).

Machine learning is a branch of artificial intelligence that focuses on making predictions by using mathematical algorithms to identify patterns in data. Deep learning is a branch of machine learning that focuses on making predictions using multi-layered neural network algorithms inspired by the neural structure of the brain. In contrast to other machine learning methods, such as logistic regression, deep learning’s neural network architecture enables models to scale exponentially as the amount and dimension of data grow (24). Machine learning algorithms to help with cancer detection (identifying the presence of cancer) and diagnosis (characterizing cancer) have become increasingly common (25, 26). In this study, we integrated scRNA-Seq data from three different samples from the Gene Expression Omnibus (GEO) database to identify cell clusters associated with PAAD occurrence. RNA-Seq data from PAAD samples and normal samples from the Cancer Genome Atlas (TCGA) and Genotype-Tissue Expression (GTEx) databases were subsequently identified by weighted gene correlation network analysis (WGCNA), which identified cellular clusters associated with gene modules, and we screened prognostic genes associated with PAAD occurrence by the univariate COX model and the least absolute shrinkage and selection operator (LASSO) COX model to construct a prognostic risk assessment system for PAAD.

## Materials and methods

### Data acquisition

The scRNA-Seq data (registration number: GSE165399) were downloaded from the GEO (https://www.ncbi.nlm.nih.gov/geo/) database, containing three samples, and the sample information is presented in **Table 1**. Four PAAD patient sequencing datasets were also downloaded (registration numbers: GSE28735, tumor samples: 42; GSE57495, tumor samples: 63; GSE62452, tumor samples: 64; and GSE85916, tumor samples: 79). RNA-Seq data (TCGA-PAAD, tumor samples: 177) from the PAAD sequencing project were downloaded from TCGA database (https://portal.gdc.cancer.gov/), as well as clinical information for the 177 samples. Normal pancreatic samples were downloaded from the GTEx (https://www.gtexportal.org/home/) database. Finally, RNA-seq data from the IGGC-AU sequencing project were downloaded from the University of California Santa Cruz (UCSC Xena, https://xena.ucsc.edu/).

**Table 1:** Clinical information for samples in the GSE165399 cohort.

Sample ID	Sample tissues	Age	Gender
GSM5032771	Intraductal papillary mucinous neoplasm	74	Male
GSM5032772	Pancreatic adenosquamous carcinoma	59	Male
GSM5032773	normal pancreas sample	50	Male

### scRNA-Seq data pre-processing

The scRNA-Seq data of the GSE165399 cohort samples were processed utilizing the Seurat package (27). First, the genes that were expressed in all three cells were screened, and the number of genes expressed in each cell was greater than 250. The PercentageFeatureSet function was employed to calculate the percentage of mitochondria and rRNA and to ensure that each cell expressed more than 500 and less than 7,000 genes with less than 30% mitochondrial content. Also, the number of UMI in each cell was ensured to be no less than 500.

### scRNA-Seq data clustering and dimension reduction

Initially, the samples were merged by the merge function in the Seurat package, and the merged data were normalized by log normalization. High-variability genes were then detected by the FindVariableFeatures function (based on the variance stabilization transformation (vst) to identify variable features). All genes were scaled with the ScaleData function and subjected to principal component analysis (PCA) with the RunPCA function. We then performed cell clustering analysis (set resolution = 0.3) by selecting dim = 40 and identifying specific cell clusters in PAAD by the FindNeighbors and FindClusters functions. Next, with the top 40 principal components selected, we operated the UMAP program to further reduce the dimensionality. Finally, we screened marker genes in cell clusters by |logfold change (FC)| = 0.35 and Minpct = 0.3 (minimum expression ratio of differential genes) via the FindAllMarkers function.

### RNA-Seq data processing

RNA-Seq data were processed on the SangerBox website, a comprehensive online bioinformatics analysis website (28). Samples without follow-up information were removed from the TCGA-PAAD cohort, FPKM data were transformed into TPM data, and normal pancreatic samples from the UCSC Xena were subsequently merged, and the merged cohort was recorded as TCGA _GTEx-PAAD (tumor: 177, normal: 167, gene number: 24210). Normal samples, samples with missing follow-up information in the GSE28735, GSE57495, GSE62452, and GSE85916 cohorts were excluded.

### Annotation of cell clusters

The cell marker genes for human cells were selected from the official cell marker website (http://biocc.hrbmu.edu.cn/CellMarker/) for the pancreas, pancreatic acinar tissue, peripheral blood, and blood corresponding tissues. The enricher function in the clusterProfiler package (29) was provided for cell cluster annotation.

### Monocle trajectory analysis

Monocle (version 2.18.0) is used to infer the developmental trajectory of subpopulations of cells. Cells were isolated from the Seurat object and transferred into the SingleCellExperiment format (follow the official tutorial for trajectory analysis: http://cole-trapnell-lab.github.io/monocle-release/docs/#constructing-single-cell-trajectories). The Monocle object is built from the SingleCellExperiment format using the new cell dataset function in Monocle.

### PAAD-related cell cluster abundance analysis

Based on marker genes in cell clusters, we computed the relative abundance of cell subpopulations in tumor and normal tissues in the TCGA_GTEx-PAAD cohort using the CIBERSORT method (30).

### WGCNA analysis

To identify key genes for tumorigenesis, we performed WGCNA analysis on samples in the TCGA_GTEx-PAAD cohort. Cluster analysis was first performed on 177 tumor samples and 167 normal samples to further calculate the Pearson’s correlation between each gene, followed by constructing co-expression networks and forming gene modules using the WGCNA package (31). Subsequently, the Pearson’s correlation analysis was performed with each gene module using the first principal component (ME) of the cell subpopulation to identify the key gene modules for PAAD occurrence. The Monocle3 package was also performed to analyze cellular pseudo-temporal trajectories (32).

### Enrichment analysis

To explore the biological functions as well as signaling pathways involved in genes within the key modules of PAAD occurrence, we performed Gene Ontology (GO) and Kyoto Encyclopedia of Genes and Genomes (KEGG) functional enrichment analyses using the clusterProfiler package at *p*< 0.05 and FDR< 0.05 as thresholds to screen the most significantly enriched molecular functions and signaling pathways.

### Cell communication analysis

In multicellular organisms, the basic vital activities of life depend on cell–cell interactions as a contribution to the coordination of their behavior. The communication between cells relies mainly on multisubunit protein complexes. Based on this, we used the cell chart package (33) to analyze the number of interacting ligands between all cell subpopulations as well as changes in the strength of the interaction.

### Screening for PAAD-generating genes

Differential analysis was conducted via the limma package (34) to obtain tumor-associated differentially expressed genes (DEGs) in tumor and normal tissues in the TCGA_GTEx-PAAD cohort, which were subsequently intersected with cellular subpopulations associated with PAAD occurrence, and genes within the signature module were taken to obtain key genes for PAAD occurrence.

### Prognostic model construction, evaluation, and validation

Univariate COX models were generated for the expression matrix of tumor samples from the TCGA-PAAD cohort in combination with patient survival status and survival time to identify genes affecting PAAD survival (*p*< 0.01). Models with a large number of genes were not conducive to clinical test manipulation, so we constructed LASSO COX models based on the above genes employing the glmnet package (35, 36) and removed genes with high similarity in the models by introducing the penalty parameter lambda in 10-fold crossvalidation. The resulting genes were the PAAD prognostic signature genes. Based on the regression coefficients in the LASSO COX model and the expression levels of individual genes, we constructed the RiskScore for the PAAD prognostic risk assessment tool, which was calculated by the following equation.


$$\text{RiskScore}=\sum^{\;}\beta_{i\;}*\text{Exp }i$$

where β was the regression coefficient normalized by the *Z*-score for each gene in the LASSO COX model, and Exp represented the gene expression data.

The RiskScore of tumor samples in the TCGA-PAAD, GSE28735, GSE57495, GSE62452, GSE85916, and IGGC-AU cohorts was determined according to the formula, and the high RiskScore group and low RiskScore group were classified based on RiskScore = 0 as the threshold. Kaplan–Meier (K-M) survival curves were plotted to assess the prognostic differences between the two groups, and receiver operating characteristic curve (ROC) was developed to assess the performance of RiskScore in predicting PAAD prognosis.

### Potential associations between RiskScore and clinical features

Patients in the TCGA-PAAD cohort were grouped according to clinical features, and the RiskScore of patients in each subgroup was counted. The Wilcox test was conducted to calculate the statistical difference between the two groups, and the Kruskal–Wallis test was conducted to calculate the statistical difference among the four groups. *P*< 0.05 was considered to be significantly distinct.

### Gene set enrichment analysis

To explore the biological pathways that existed differently between the high RiskScore and low RiskScore groups, single sample gene set enrichment analysis (ssGSEA) was performed on the high RiskScore samples and low RiskScore samples in the TCGA-PAAD cohort, and the ssGSEA scores of pathways were analyzed for the Pearson’s correlation with RiskScore, and pathways with *r* > 0.5 were considered potentially regulated pathways by RiskScore.

### Endocrine metabolism analysis

The KEGG website provided 34 secretory genes. Genes in SECRETORY_PATHWAY were obtained from the GSEA website (https://www.gsea-msigdb.org/gsea/index.jsp) to calculate the score using ssGSEA.

### Statistical analysis

This study was performed using R software (version 4.1.1) for data analysis. For all statistical analyses, bilateral *p*< 0.05 was considered statistically significant.

## Results

### Dimensionality reduction and clustering of scRNA-Seq data

**Figure 1:**
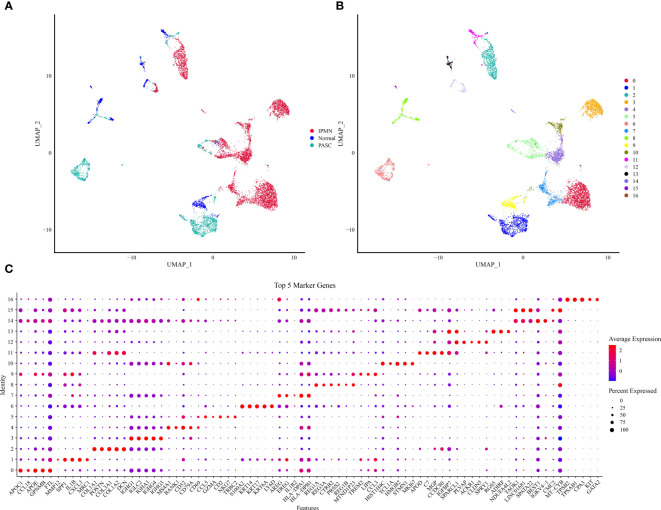
UMAP downscaling analysis of scRNA data. **(A)** Distribution of cell subpopulations in GSM5032771, GSM5032772, and GSM5032773 samples. **(B)** Distribution of 17 cell clusters. **(C)** Heatmap of top 50 gene expression in 17 cell clusters.

Initially, the scRNA-Seq data were filtered to retain the genes that were expressed in GSM5032771, GSM5032772, and GSM5032773 (**Supplementary Figures S1**, **S2**). The filtered data were combined, and the highly variable genes in the samples were filtered by the FindVariableFeatures function. The volcano figure showed the highly variable genes in the samples and marked the top 20 highly labeled genes (**Supplementary Figure S3**). All genes were scaled using the ScaleData function, and PCA downscaling was performed to find the anchor points (**Supplementary Figure S4**). By cluster analysis, we obtained 17 subgroups and showed their distribution characteristics in the sample (**Figure 1A**), and we further selected the top 40 principal components to further downscale by UMAP to obtain 17 cell clusters (**Figure 1B**). We used the FindAllMarkers function to screen marker genes in 17 clusters by |logFC| = 0.35, Minpct = 0.3 (minimum expression proportion of difference genes) with corrected *p*< 0.05, and **Figure 1C** demonstrates the top five marker gene expression levels in 17 cell clusters.

### Annotation of 17 cell clusters

**Figure 2:**
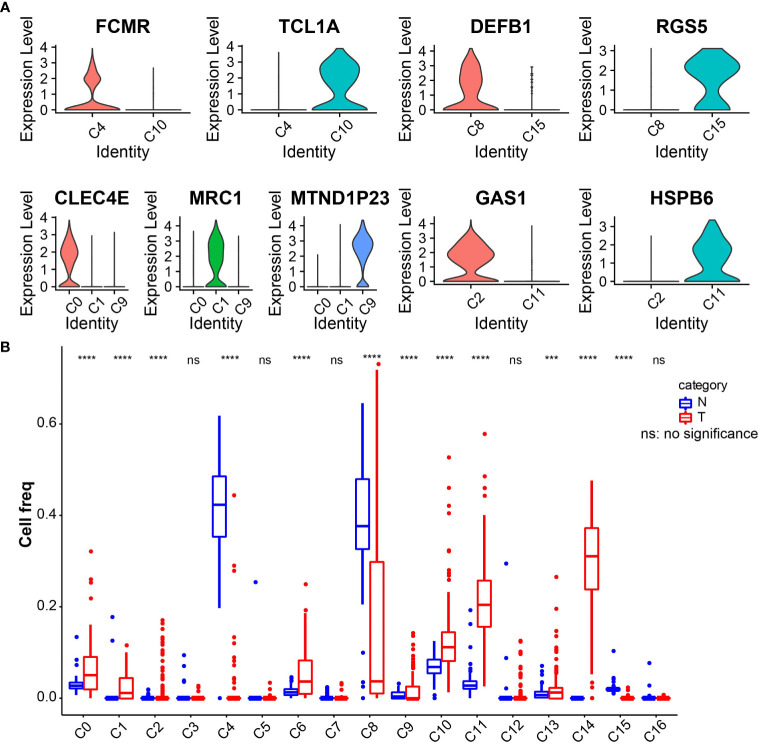
**(A)** Violin plot of expression of characteristic genes in cell clusters. **(B)** Boxplot of the abundance of 17 cell clusters in tumor and normal tissues in the TCGA_GTEx-PAAD cohort. ***p<0.001, ****p<0.0001.

The marker genes of the human pancreas, pancreatic acinar tissue, peripheral blood, and blood tissues were annotated by the enricher function of the clusterProfiler package for 17 cell clusters. The annotation information of each cell cluster is shown in **Table 2**. We found the presence of multiple small clusters in four cell subgroups, including B cell with two clusters, C4 and C10; cancer cell with two clusters, C8 and C15; CD1C–CD141-dendritic cell with three clusters, C0, C1, and C9; and fibroblast with C2 and C11 clusters. Furthermore, we analyzed the differential expression of marker genes in each cell cluster, and we found that C0 specifically expressed CLEC4E, C1 specifically expressed MRC1, C2 subpopulation specifically expressed GAS1, C4 specifically expressed FCMR, C8 specifically expressed DEFB1, C9 specifically expressed MTND1P23, C10 specifically expressed TCL1A, C11 specifically expressed the *HSPB6* gene, and C15 specifically expressed RGS5 (**Figure 2A**). In addition, we found higher abundances of C0, C1, C2, C4, C6, C8, C9, C10, C11, C13, C14, and C15 in tumor tissues (**Figure 2B**).

**Table 2:** Annotation information for 17 cell clusters.

Seraut_cluster	Cell_type
C0	CD1C-CD141- dendritic cell
C1	CD1C-CD141- dendritic cell
C2	Fibroblast
C3	Plasmacytoid dendritic cell
C4	B cell
C5	T cell
C6	Epithelial cell
C7	CD1C+_B dendritic cell
C8	Cancer cell
C9	CD1C-CD141- dendritic cell
C10	B cell
C11	Fibroblast
C12	Endothelial cell
C13	CD141+CLEC9A+ dendritic cell
C14	Circulating fetal cell
C15	Cancer cell
C16	Basophil

### PAAD-related gene module identification

**Figure 3:**
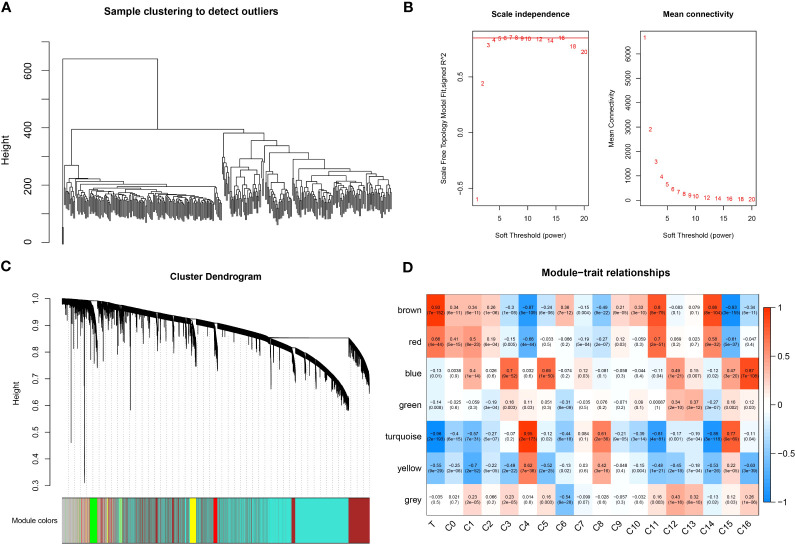
WGCNA analysis. **(A)** Sample clustering map. **(B)** Soft threshold β selection in a scale-free network. **(C)** Gene modules. **(D)** Correlation heatmap.

**Figure 4:**
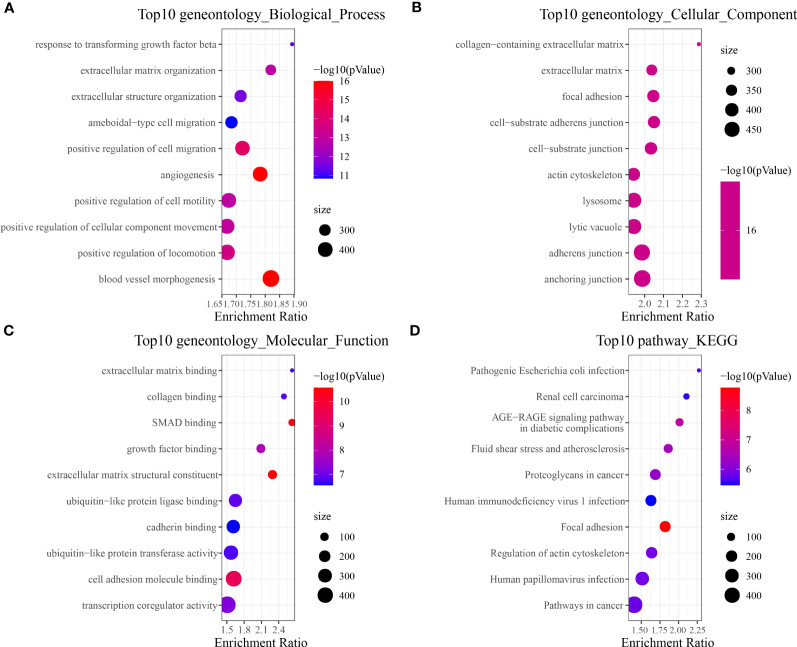
Brown module gene function enrichment analysis. **(A)** Biological process. **(B)** Cellular component. **(C)** Molecular function. **(D)** KEGG.

To identify tumor-associated gene modules in PAAD, we performed a WGCNA analysis. After the samples were clustered to construct a scale-free network, we found that the co-expression network conformed to the scale-free network at the soft threshold β = 7, when the scale-free *R*^2^ was 0.85 (**Figures 3A–C**). A total of six gene modules were generated, among which the brown module (gene number: 4811) was highly correlated with the C11 (*r* = 0.8, *p* = 5e−79) and C14 (*r* = 0.86, *p* = 8e−104) cluster (**Figure 3D**). To explore the biological functions of genes within the brown module, we performed GO and KEGG enrichment analyses. We found that these genes were mainly involved in biological processes like angiogenesis and blood vessel morphogenesis; they may also be involved in extracellular matrix and extracellular matrix that contains collagen, adherens junctions between cells and their substrates, and focal adhesion sites; they are also closely related to SMAD binding, extracellular matrix structural constituents, and cell adhesion molecule-binding functions (**Figures 4A–C**). We also revealed that these genes are actively involved in signaling pathways such as focal adhesion and regulation of the actin cytoskeleton (**Figure 4D**). Our results suggested that genes within the brown module were intimately associated with intercellular signaling transitions.

### Trajectory analysis of critical cell cluster

**Figure 5:**
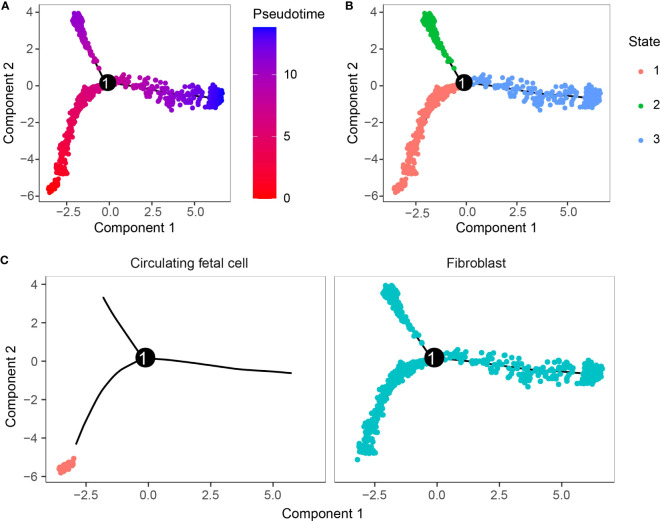
Cell trajectory analysis. **(A)** Pseudo-time measurement of developmental time. **(B)** Two cell subpopulations could differentiate into three branches. **(C)** Differentiation trajectory of cell clusters.

The WCGNA analysis revealed a significant correlation between genes within the brown module and tumorigenesis, while the module was highly correlated with the C11 and C14 clusters. The two clusters belong to fibroblast and circulating fetal cells, respectively, where fibroblast cells are characterized by two clusters, C11 and C2. We suggested that the two clusters might be critical clusters for tumorigenesis. We then performed cell trajectory analysis of the critical cluster by Monocle. From the cell differentiation trajectory, C11 and C2, which were also fibroblast cells, showed the same differentiation trend, basically at the tail end of the state 1 branch (**Figures 5A, B**), while the heterogeneity of circulating fetal cell cells was extremely high (**Figure 5C**).

### Cell communication analysis

**Figure 6:**
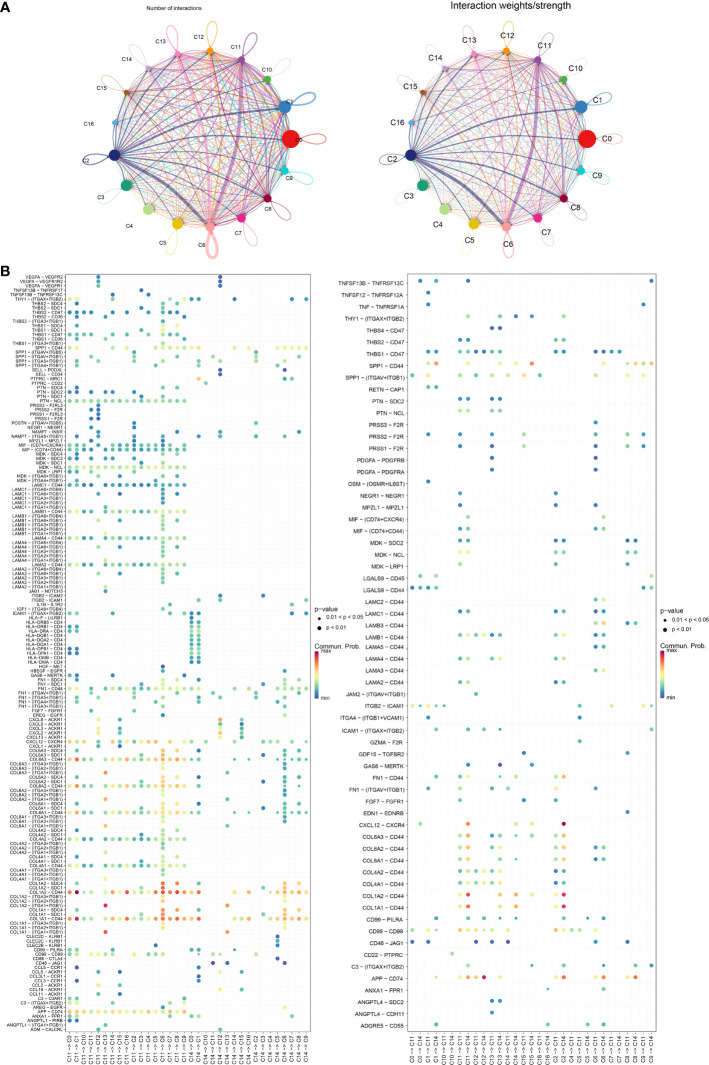
**(A)** Graph of changes in the number of receptors and ligands as well as intensity in cellular communication of 17 cell clusters. **(B)** Bubble diagram of receptors and ligands of C11 and C14 cell clusters with other cell clusters.

To better investigate how the C11 cluster communicates with other cell clusters, we performed a cellular communication analysis. **Figure 6A** shows the interactions and intensity changes between 17 cell clusters, and the results indicate a high correlation between cells. Subsequently, we extracted the ligand–receptor information of each subpopulation to communicate with each other, and we found that C11 and C14 influenced another cluster through some ligand receptors, and the C11 subpopulation influenced other cell cluster by acting on cell surface receptors through LAMC1 (**Figure 6B**). In addition, we also found some novel pairing relationships, such as LAMA4-CD44 and FN1-SDC4, and these results suggested that the C11 subpopulation played a great role in the development of PAAD.

### Tumorigenesis gene screening

**Figure 7:**
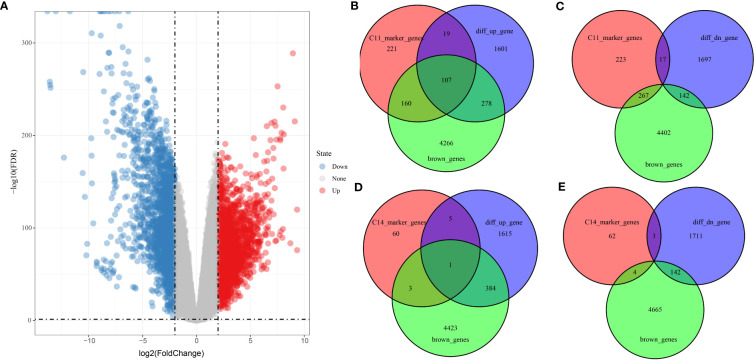
Screening of genes related to PAAD occurrence. **(A)** Volcano plot of DEGs between tumor and normal tissues in TCGA_GTEx-PAAD cohort. **(B**, **C)** Wayne plots of genes within C11 cell clusters, up- and downregulated DEGs, and brown modules. **(D**, **E)** Wayne plots of genes within C14 cell clusters, up- and downregulated DEGs, and brown modules.

Differential analysis of tumor samples and normal samples in TCGA_GTEx-PAAD identified 3,864 DEGs in tumor tissues, of which 2,008 upregulated DEGs and 1,856 downregulated DEGs were identified (**Figure 7A**). Furthermore, Venn diagrams were drawn to identify overlapping genes in DEGs, brown module genes, and marker genes in C11 and C14 cell clusters. The C11 cluster, DEGS, and brown module genes contained 107 overlapping genes (**Figures 7B, C**), while the C14 cluster, DEGs, and brown module genes contained one overlapping gene (**Figures 7D, E**). Our results indicated that the overlapping genes were all highly expressed in tumor tissues. Only one overlapping gene was present in marker genes in the C14 cluster; therefore, we concluded that genes in the C11 cluster might be pivotal genes in PAAD tumorigenesis.

### PAAD clinical prognostic model

**Figure 8:**
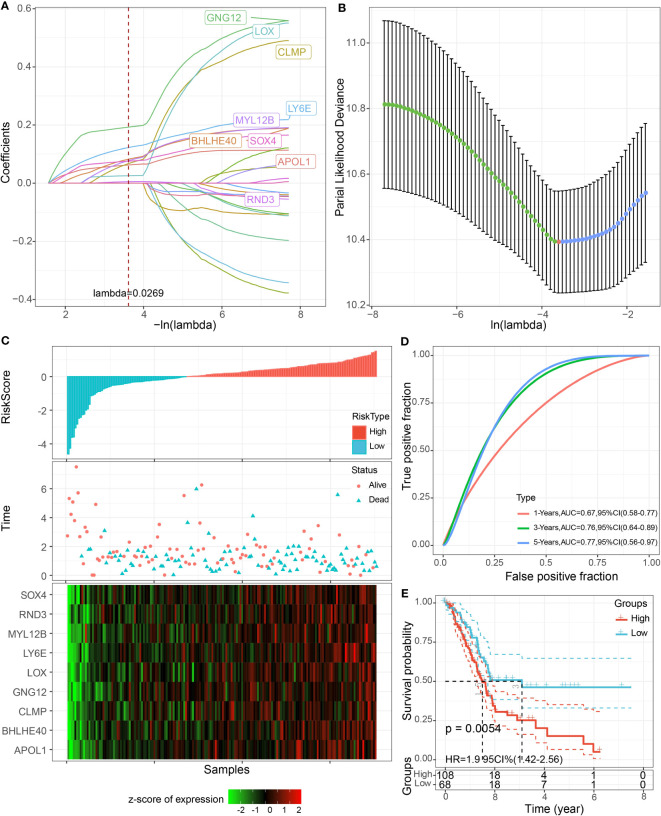
LASSO COX model construction. **(A)** Trajectory plot of independent variables with lambda. **(B)** The confidence interval of lambda. **(C)** Scatter plot of RiskScore distribution, survival status, and nine-gene expression heatmap of patients in TCGA-PAAD cohort. **(D)** ROC curves. **(E)** K-M curves of patients in high and low RiskScore groups.

The univariate COX model found 24 prognostic genes that were significantly associated with PAAD prognosis. It was well known that multigene models were unfavorable for clinical detection, so we employed the LASSO COX model to compress the number of genes in the model and remove the genes with high similarity. Based on 10-fold crossvalidation to select the best penalty parameter lambda, we found that the model was optimal at lambda = 0.0269, so we selected nine genes (APOL, BHLHE40, CLMP, GNG12, LOX, LY6E, MYL12B, RND3, SOX4) at lambda = 0.0269 as the target genes of the next procedure (**Figures 8A, B**). Based on the regression coefficients and gene expression levels, we constructed a clinical prognosis assessment system for PAAD patients with RiskScore = 0.128 * APOL1 + 0.153 * BHLHE40 − 0.552 * CLMP − 0.363 * GNG12 + 0.528 * LOX − 0.202 * LY6E − 0.202 * MYL12B + 0.051 * RND3 + 1.003 * SOX4. Patients were classified into the high RiskScore group (*N* = 108) and low RiskScore group (*N* = 68) by RiskScore Z-score normalized to 0 as the grouping threshold for the sample. We identified that patients in the high RiskScore group had a worse prognosis and a higher death rate in the TCGA-PAAD cohort (**Figures 8C, D**). The AUC values for RiskScore to predict 1-, 3-, and 5-year survival in PAAD were 0.67, 0.76, and 0.77, respectively (**Figure 8E**).

### Validation of RiskScore

**Figure 9:**
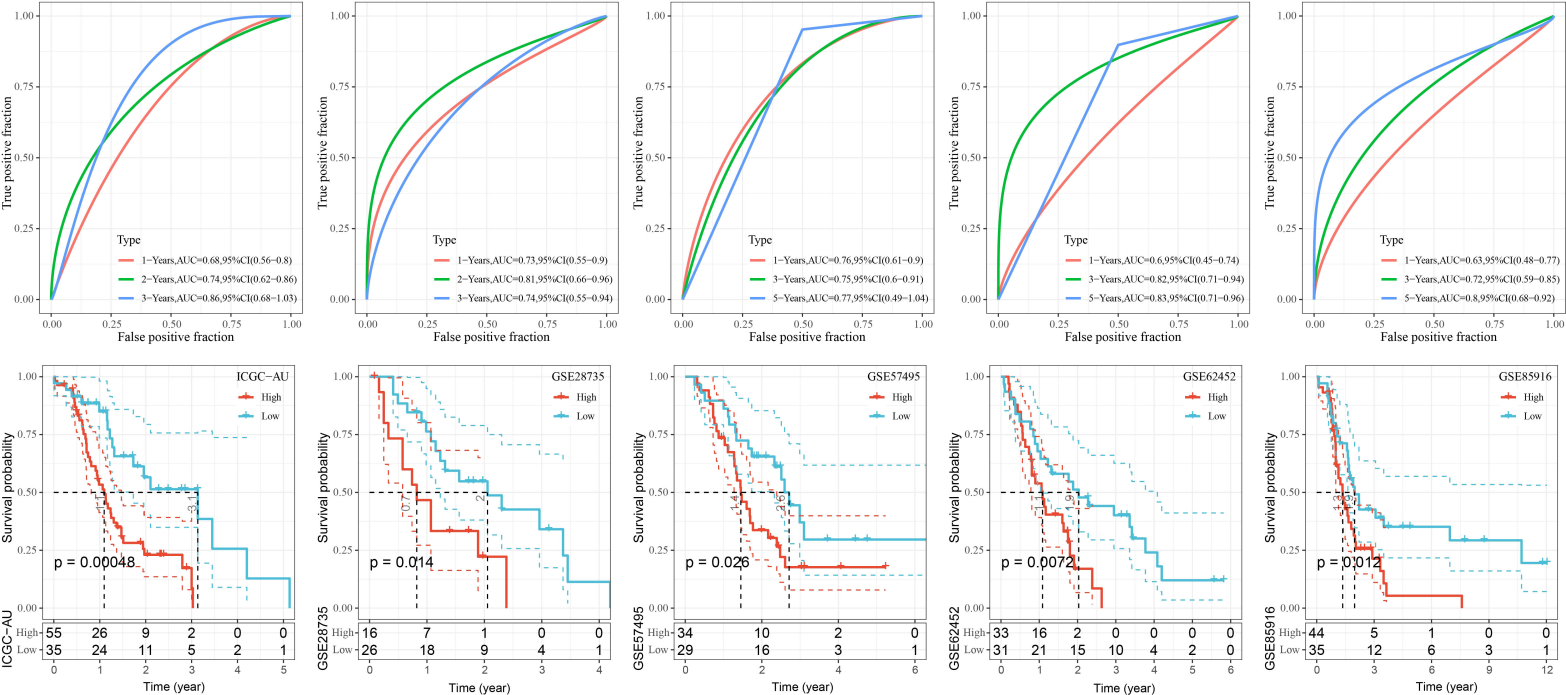
K-M curves as well as ROC curves for patients in the high and low RiskScore groups in the GSE28735, GSE57495, GSE62452, and GSE85916, ICGC-AU cohorts.

To better assess the robustness of RiskScore, the prognostic value of RiskScore was evaluated in the external datasets GSE28735, GSE57495, GSE62452, GSE85916, and ICGC-AU. We found that the OS of high RiskScore in the five datasets was significantly worse than that of the low-risk group (*p*< 0.05), and the 1-, 3-, and 5-year survival rates of RiskScore-predicting PAAD were all above 0.6 (**Figure 9**).

### Association between RiskScore and clinical features of PAAD

**Figure 10:**
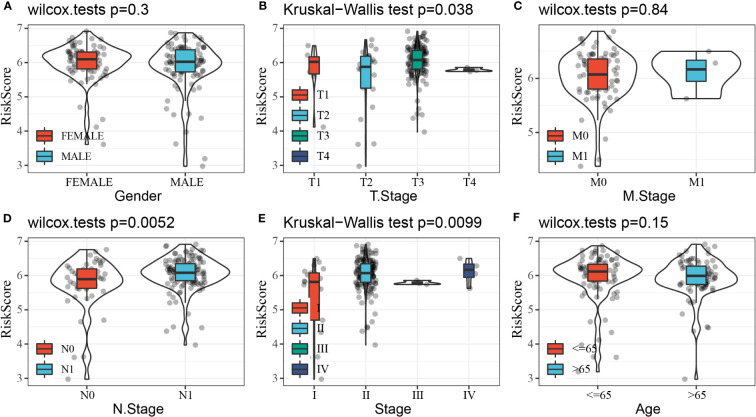
Distribution of RiskScore in subgroups of clinical features. **(A)** Gender. **(B)** T stage. **(C)** M stage. **(D)** N stage. **(E)** Stage. **(F)** Age.

Clinical features, as traditional prognostic elements, were associated with the survival rate of cancer patients. In this study, we counted the distribution of RiskScore in patients with different clinical feature subgroups. We found a significant difference between RiskScore and T stage, N stage, and stages I–IV (*p<* 0.05), and the overall trend of increasing RiskScore with increasing stage. There was no significant difference between RiskScore and gender, M, stage, and age (**Figure 10**).

### Gene set enrichment analysis

**Figure 11:**
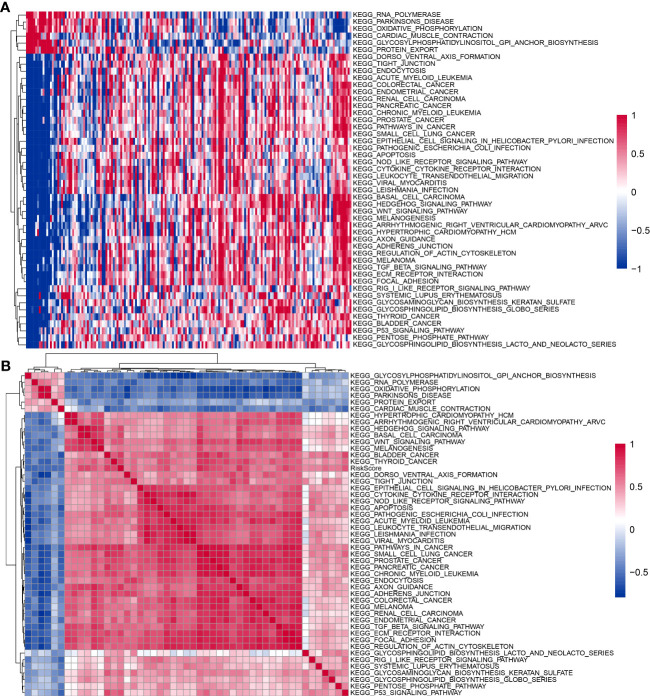
KEGG pathways affected by RiskScore. **(A)** Heatmap of clustering between 48 KEGG pathways and RiskScore. **(B)** Heatmap of KEGG pathways with changes in RiskScore.

To further investigate the relationship between RiskScore and biological function in different samples, ssGSEA enrichment analysis was performed for patients in the high and low RiskScore groups in the TCGA-PAAD cohort. Also, the Pearson’s correlation analysis was performed between the ssGSEA score of each pathway and RiskScore; a total of 48 KEGG pathways were significantly correlated with RiskScore (correlation ≥ 0.5), among which six KEGG pathways were significantly negatively correlated with RiskScore, containing KEGG_RNA_ POLYMERASE, KEGG_PARKINSONS_DISEASE, KEGG_OXIDATIVE_PHOSPHORYLATION, KEGG_CARDIAC_MUSCLE_CONTRACTION, KEGG_GLYCOSYLPHOSPHATIDYLINOSITOL_GPI_ANCHOR_BIOSYNTHESIS, and KEGG_PROTEIN_EXPORT. RiskScore was strongly and positively correlated with 42 KEGG pathways (**Figure 11A**). Subsequent cluster analysis of the samples according to each KEGG pathway revealed that KEGG_BASAL_TRANSCRIPTION_FACTORS and KEGG_PROGESTERONE_MEDIATED_OOCYTE_MATURATION pathways increased with higher RiskScore scores (**Figure 11B**).

### Immune microenvironment analysis

**Figure 12:**
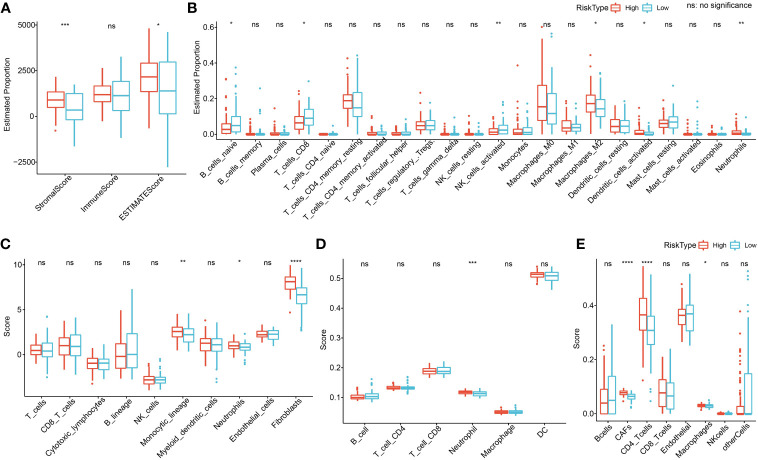
Immune microenvironment analysis. **(A)** ESTIMATE analysis. **(B)** CIBERSORT analysis shows a difference of 22 immune cells between high and low groups. **(C)** Using MCP-counter, we found 10 immune cell differences between high and low groups. **(D)** Using TIMER, six immune cell differences were found between high and low groups. **(E)** Using EPIC analysis, we found eight immune cell differences between high and low groups. *p<0.05, **p<0.01, ***p<0.001, ****p<0.0001.

To clarify the relationship between RiskScore and patients’ immune microenvironment, we first used ESTIMATE to evaluate immune infiltration. The high-risk group had a higher StromalScore and ESTIMATEScore (**Figure 12A**). CIBERSORT analysis showed that the low-risk group had significantly enriched T_cells_CD8, NK_cells_activated, and B_cells_naive, and the high-risk group had significantly enriched Macrophages_M2 (**Figure 12B**). MCP-counter, TIMER, and EPIC analyses suggested that the high-risk group had higher immune infiltration (**Figures 12C–E**).

### Endocrine metabolism analysis

**Figure 13:**
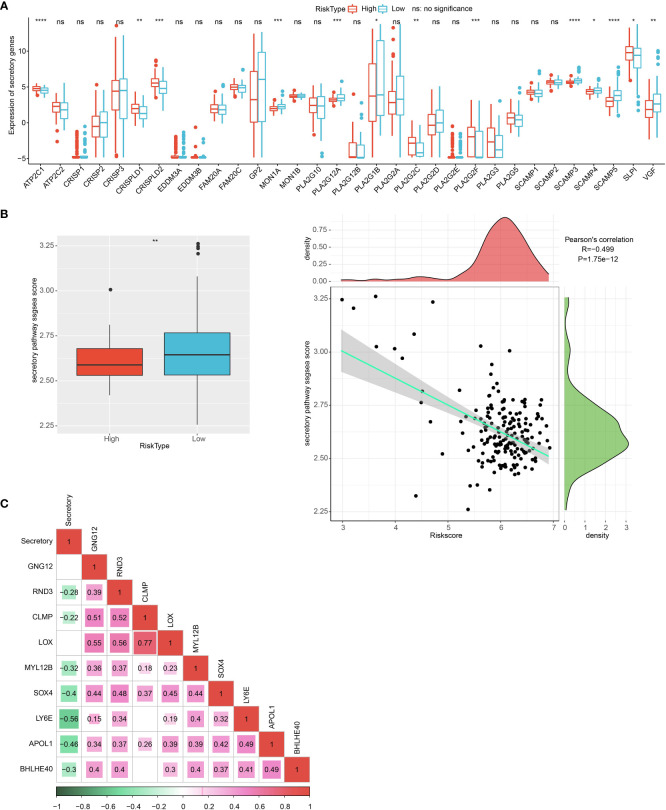
Endocrine metabolism analysis. **(A)** Difference of 34 endocrine-related genes between high and low group. **(B)** The difference in endocrine pathway scores between high and low groups. **(C)** The correlation analysis between genes in the prognosis model and the endocrine pathway score. *p<0.05, **p<0.01, ***p<0.001, ****p<0.0001.

The 13 of 34 endocrine-related gene expressions differed in the high- and low-risk groups (**Figure 13A**). The low RiskScore group had a higher secretory pathway score. In addition, a negative phenomenon was observed between the secretory pathway score and the RiskScore (**Figure 13B**). Seven genes from the prognosis model were negatively correlated with the secretory pathway score (**Figure 13C**).

### Construction of nomogram

**Figure 14:**
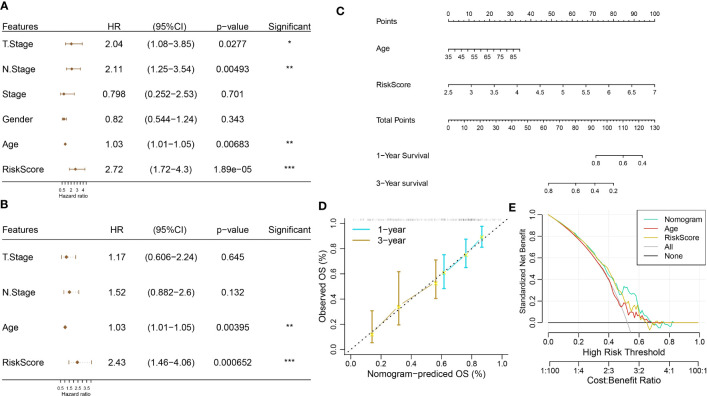
Construction of a nomogram. **(A, B)** Univariate and multivariate Cox regression analyses. **(C)** Construction of nomogram using age and RiskScore. **(D)** Calibration curve analysis. **(E)** Decision curve analysis (DCA).

Univariate and multivariate regression analyses showed that age and RiskScore were independent prognostic factors (**Figures 14A, B**). We next combined age and RiskScore to build a nomogram, which could predict the prognosis of pancreatic cancer patients (**Figure 14C**). The nomogram shows that the 1- and 3-year prognosis lines are close to the 45° standard line, indicating good predictive performance (**Figure 14D**). The decision curve analysis (DCA) was employed to further confirm the clinical effectiveness of the nomogram, followed by RiskScore and age (**Figure 14E**).

## Discussion

In this study, we integrated PAAD scRNA-Seq data as well as RNA-Seq data to construct a promising prognostic tool (RiskScore) using genes associated with PAAD tumorigenesis in fibroblast and validated the generalizability of RiskScore with multiple datasets. We also explored the correlation between KEGG pathways significantly associated with RiskScore and clinical features.

Recent years have demonstrated that scRNA-Seq sequencing technology displays powerful advantages in probing the mechanism of tumorigenesis. Firstly, we identified 17 cell clusters with specific marker expression in intraductal papillary mucinous neoplasm, pancreatic adenosquamous carcinoma, and normal pancreas samples. In our study, we indicated that the C11 subpopulation belongs to fibroblast at the stage of tumor development. CAFs were the most abundant components of the tumor microenvironment and were heterogeneous, playing a pro- or anticancer role in different individual settings (23, 37). CAFs positively influenced cancer progression in tumors by mimicking or dominating the extracellular matrix (ECM) and thus remodeling the ECM structure. For one, the remodeled ECM structure served as a physical barrier for the infiltration of immune cells with killing functions, enhancing tumor killing, and for another, the ECM served as a structural scaffold for the interaction between tumor cells and stromal cells in the TME, promoting cardiac angiogenesis to regulate tumor metastasis (38). In this study, we also identified C11 subpopulation-related gene modules mainly associated with components or biological processes such as intercellular information exchange. Thus, it was the close communication between CAFs and tumor cells that might be responsible for the development of PAAD.

In this study, we also found that the C11 cluster specifically expresses HSPB6, which is currently focused on bladder urothelial carcinoma (BLCA). High HSPB6 expression was the critical factor for BLCA cell migration, and elevated HSPB6 expression inhibited BLCA cell migration (39). In contrast, the results of cell communication analysis demonstrated that the C11 cluster could be influenced by other cells by interacting with cell surface receptors via LAMC1. LAMC1 secretion was associated with the formation of inflammatory CAFs in esophageal squamous cell carcinoma, and upregulation of LAMC1 expression promoted CXCL1 secretion, which stimulated inflammatory CAFs via CXCR2-pSTAT3 and thus promoted tumor progression (40). Trajectory analysis showed consistent differentiation trends between the C11 and C2 clusters in fibroblasts, but C2 was not a tumorigenesis-associated cell cluster, and the distinction was that the specifically characterized genes were different, whereas the mechanism of HSPB6 in PAAD was unknown and its function in fibroblast was unclear. Our findings provided a new potential mechanism by which the C11 cluster-specific expression of HSPB6 may promote PAAD development.

We constructed the RiskScore tool to attempt to assess the prognosis of PAAD patients. We calculated the RiskScore based on the formula, and PAAD patients were divided into a high RiskScore group and a low RiskScore group. The results indicated that the RiskScore demonstrated a good prognostic value, and patients in the high RiskScore group had a worse prognosis. This result was validated in all five external datasets. We also performed ssGSEA analysis on samples from the high and low RiskScore groups, and basal transcription factors and progesterone-mediated oocyte maturation pathways were the characteristic pathways in the high RiskScore group. Moreover, RiskScore is negatively correlated with endocrine pathways, and the high-risk group had an enhanced immune infiltration status.

RiskScore consisted of APOL1, BHLHE40, CLMP, GNG12, LOX, LY6E, MYL12B, RND3, and SOX4, all of which were PAAD prognosis-associated genes. APOL1 was observed to be a critical enzyme in lipid functioning and metabolic processes and was found to be aberrantly highly expressed in hepatocellular carcinoma, small-cell lung cancer, and bladder cancer (41–44). Recent studies indicated that APOL1 exhibited oncogenic effects in PAAD, inhibiting PAAD cell apoptosis and promoting tumor cell proliferation through activation of the NOTCH1 signaling pathway (45), which was the first study reporting APOL1 function in PAAD. Overexpression of BHLHE40 caused the differentiation of tumor-associated neutrophils into a protumor subpopulation (TAN-1) and enhanced tumor immune suppression (46). CLMP was the central immune-related gene in colon cancer, associated with the inflammatory response, KRAS signaling pathway, and T-cell infiltration (47). Upregulation of pro-oncogenic MiR-106b-5p expression influenced survival outcomes in invasive breast cancer via suppression of GNG12 (48). LOX family genes were remodeling agents of hypoxia-induced ECM and were also pivotal inducers of chemotherapeutic drug resistance (49). The remaining genes were also found to positively influence cancer progression (50–53). CLMP, GNG12, LOX, LY6E, MYL12B, and SOX4 were reported for the first time as prognostic signature genes for PAAD, and the mechanisms of how they regulate PAAD occurrence deserve further investigation.

Our study defined the critical cell cluster during PAAD genesis, which might promote tumor progression through frequent communication with tumor cells. In addition, we constructed a robust prognostic tool that demonstrated good robustness in predicting PAAD prognosis. However, this study was a comprehensive bioinformatic analysis conducted with public databases, and the molecular mechanisms of the C11 cluster and PAAD prognostic genes still remain to be further confirmed by relevant experiments as well as clinical trials.

## Conclusion

In conclusion, we identified the C11 cluster in fibroblasts that specifically expressed HSPB6 as the essential cluster for PAAD development and constructed a nine-gene prognostic model through tumor-associated PAAD prognostic genes in the C11 subpopulation. RiskScore might carry a credible clinical prognostic potential for PAAD.

## Data availability statement

All data generated or analyzed during this study are included in this published article.

## Author contributions

All authors contributed to this present work: YX and XC designed the study, and NL acquired the data. QW drafted the manuscript, and YX revised the manuscript. All authors read and approved the manuscript.

## Supplementary material

Supplementary Figure 1 Gene quality control map in the sample before filtering.

Supplementary Figure 2 Gene quality control plot in the sample after filtering.

Supplementary Figure 3 Distribution of high variant genes and non-high variant genes, the left panel showed the distribution of high variant genes and the right panel showed the distribution of non-high variant genes.

Supplementary Figure 4 PCA downscaling to find anchor points The left figure showed the anchor points in GSM5032771, GSM5032772, GSM5032773, and the right figure showed all the principal components in PCA analysis.
