# Supplementary figures and images for: Identification of fibroblast-related genes based on single-cell and machine learning to predict the prognosis and endocrine metabolism of pancreatic cancer
Source: Front Endocrinol (Lausanne). 2023 Jul 31;14:1201755. doi: 10.3389/fendo.2023.1201755 (PMC10425556; doi:10.3389/fendo.2023.1201755)

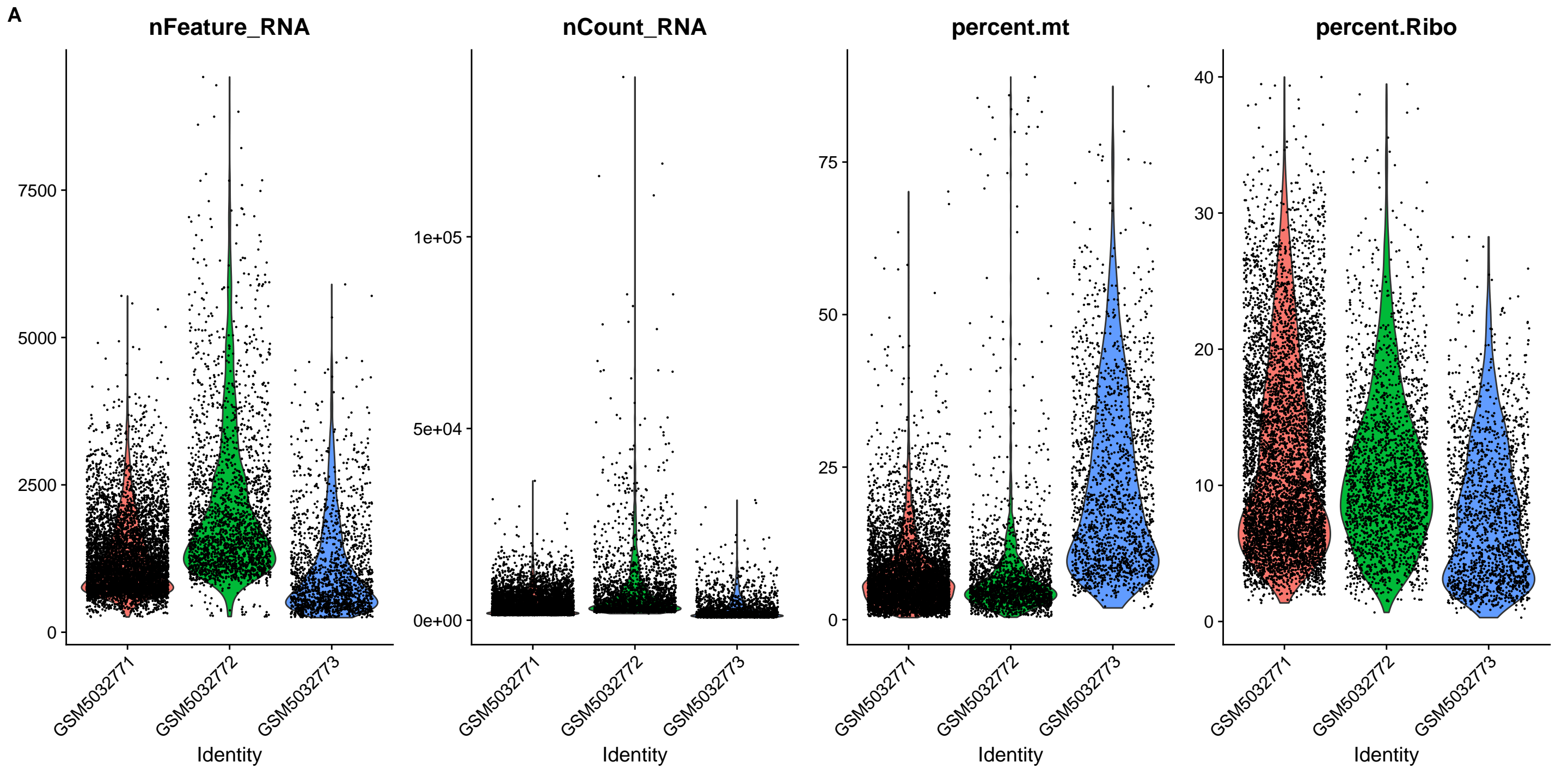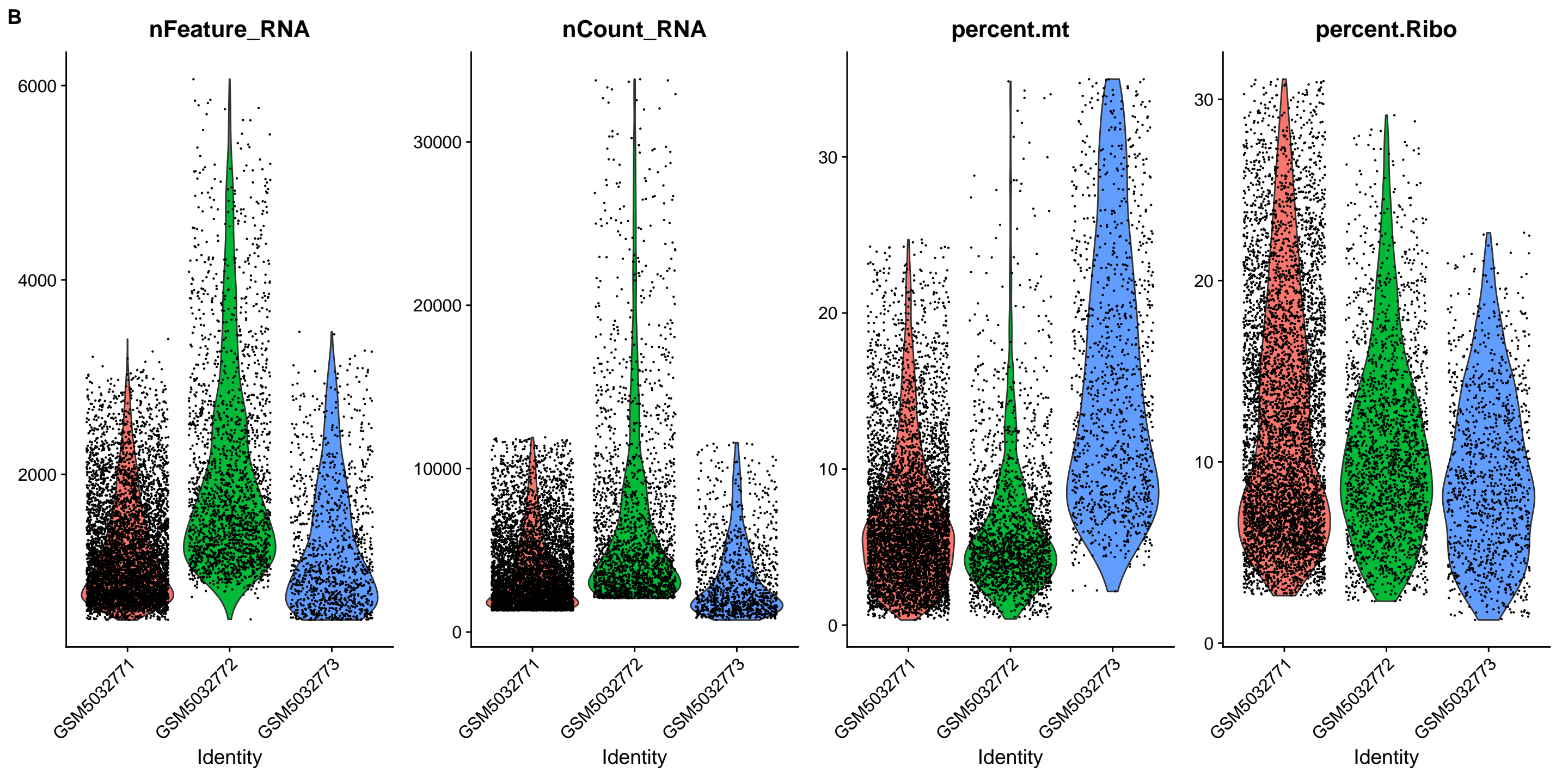

Supplement: Supplementary Figure 1 — Gene quality control map in the sample before filtering. [file DataSheet_1.pdf]

GSM5032771

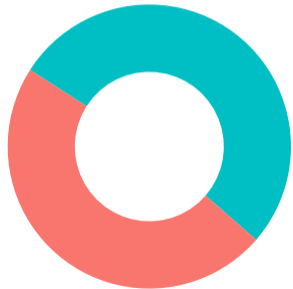

GSM5032772

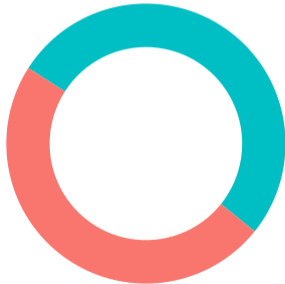

GSM5032773

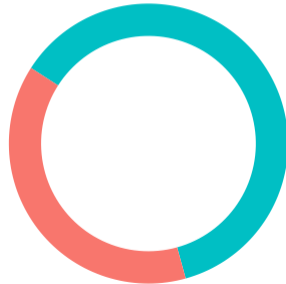

Stat

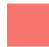

clean

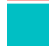

raw

Supplement: Supplementary Figure 2 — Gene quality control plot in the sample after filtering. [file DataSheet_2.pdf]

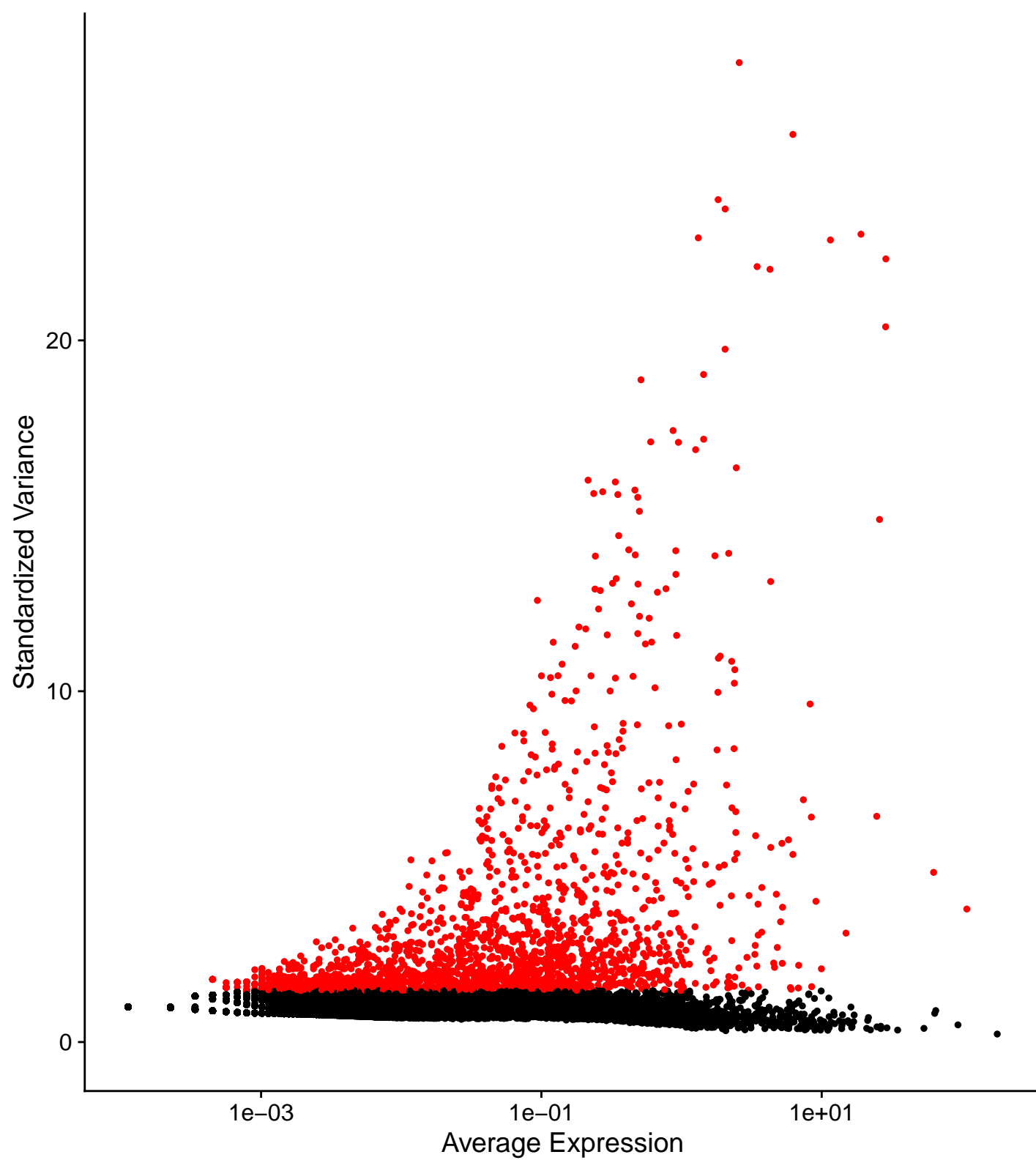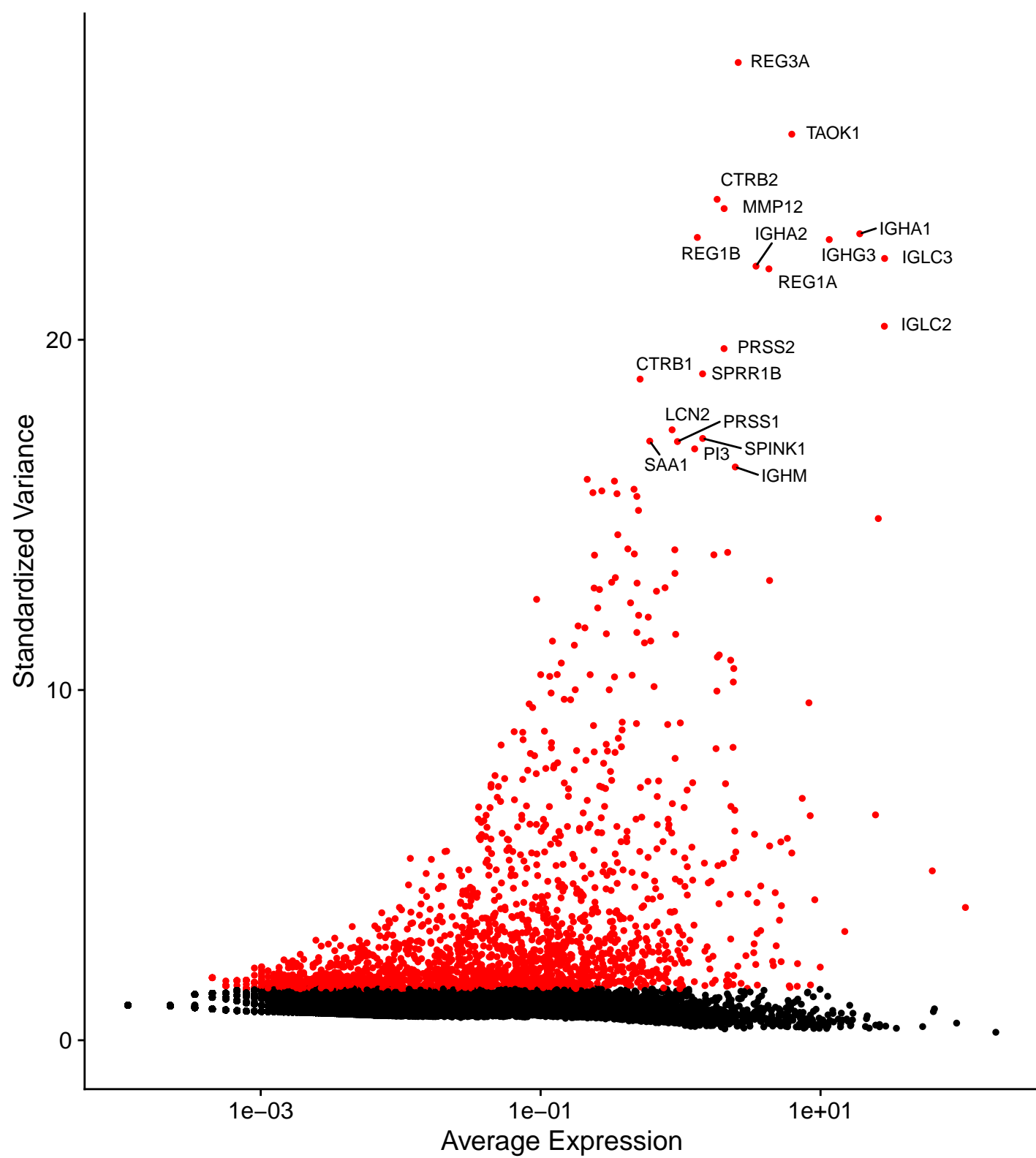

• Non-variable count: 23353 • Variable count: 2000

Supplement: Supplementary Figure 3 — Distribution of high variant genes and non-high variant genes, the left panel showed the distribution of high variant genes and the right panel showed the distribution of non-high variant genes. [file DataSheet_3.pdf]

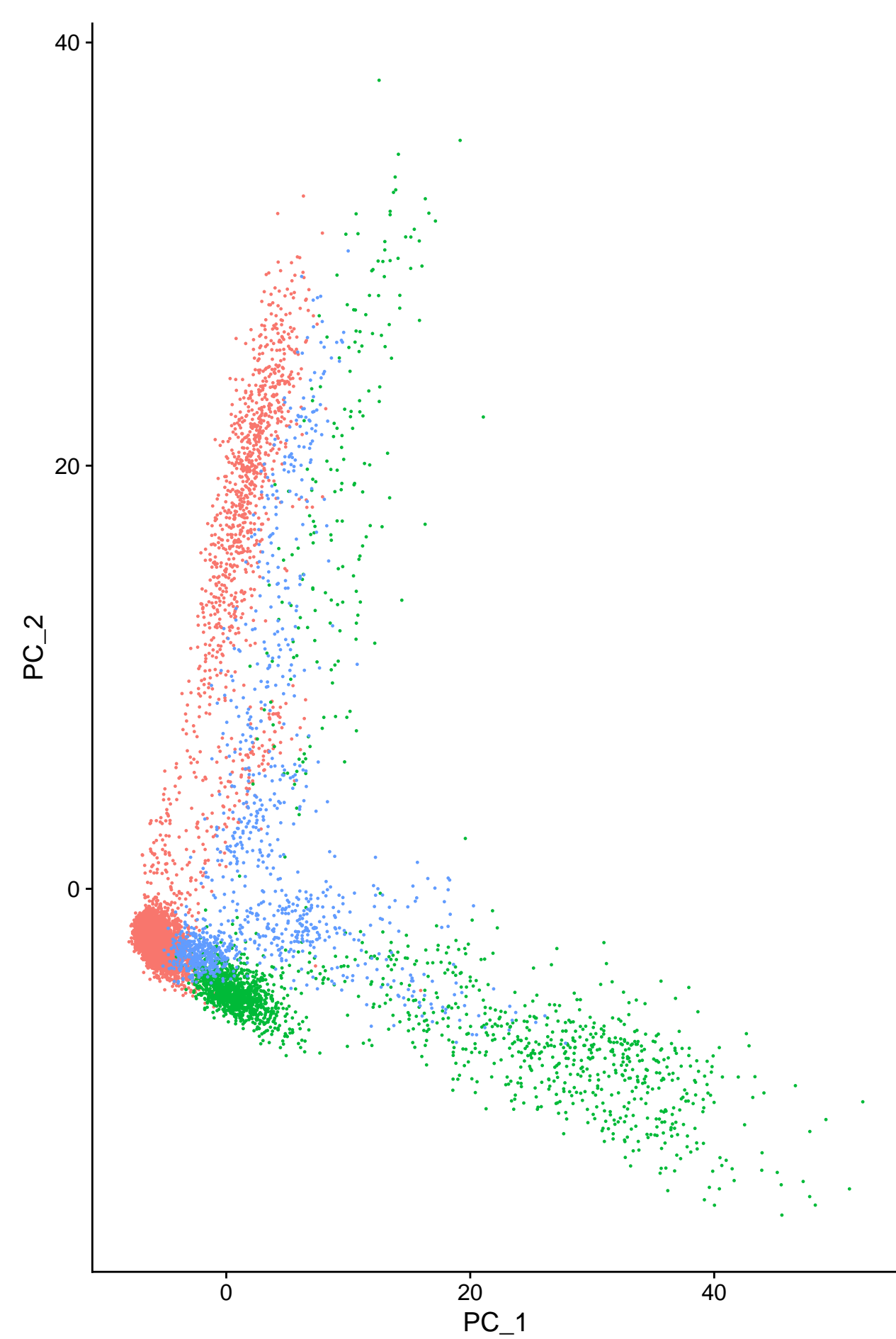

● GSM5032771  
● GSM5032772  
● GSM5032773

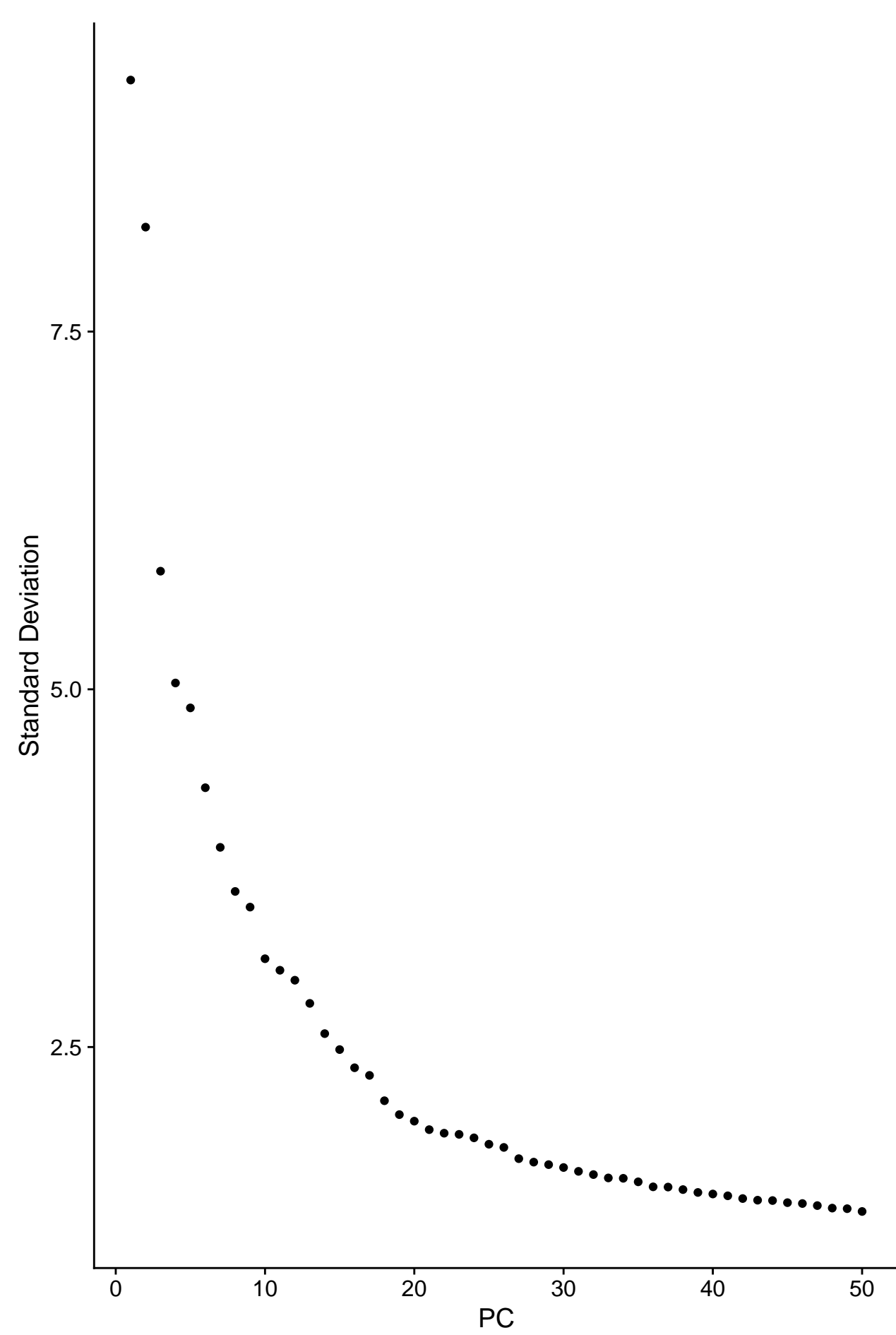

Supplement: Supplementary Figure 4 — PCA downscaling to find anchor points The left figure showed the anchor points in GSM5032771, GSM5032772, GSM5032773, and the right figure showed all the principal components in PCA analysis. [file DataSheet_4.pdf]
